# Diagnostic imaging of intrathoracic extramedullary haematopoiesis

**DOI:** 10.1002/rcr2.1212

**Published:** 2023-09-05

**Authors:** Kenichiro Takeda, Yuki Yato, Mikihito Saito, Go Saito, Akira Nishiyama, Hajime Kasai, Takuji Suzuki

**Affiliations:** ^1^ Department of Respirology, Graduate School of Medicine Chiba University Chiba Japan; ^2^ Health Professional Development Center Chiba University Hospital Chiba Japan; ^3^ Department of Radiology, Graduate School of Medicine Chiba University Chiba Japan; ^4^ Department of Medical Education, Graduate School of Medicine Chiba University Chiba Japan

**Keywords:** bone marrow scintigraphy, extramedullary haematopoiesis, pleural nodules, pulmonary nodules

## Abstract

Although intrathoracic extramedullary haematopoiesis (EMH) is rare, its nodular lesions should be differentiated from malignancy. ^111^In‐bone marrow scintigraphy can be useful for the non‐invasive diagnosis of intrathoracic EMH because extramedullary accumulation of ^111^In can be determined as EMH.

## Graphical abstract

Intrathoracic extramedullary haematopoiesis (EMH) can present as nodular lesions that need to be differentiated from malignancy. ^111^In‐bone marrow scintigraphy can be useful for the non‐invasive diagnosis of intrathoracic EMH because the extramedullary accumulation of ^111^In can be determined as EMH.

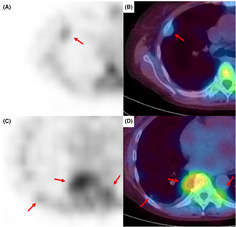

## CLINICAL IMAGE

**FIGURE 1:**
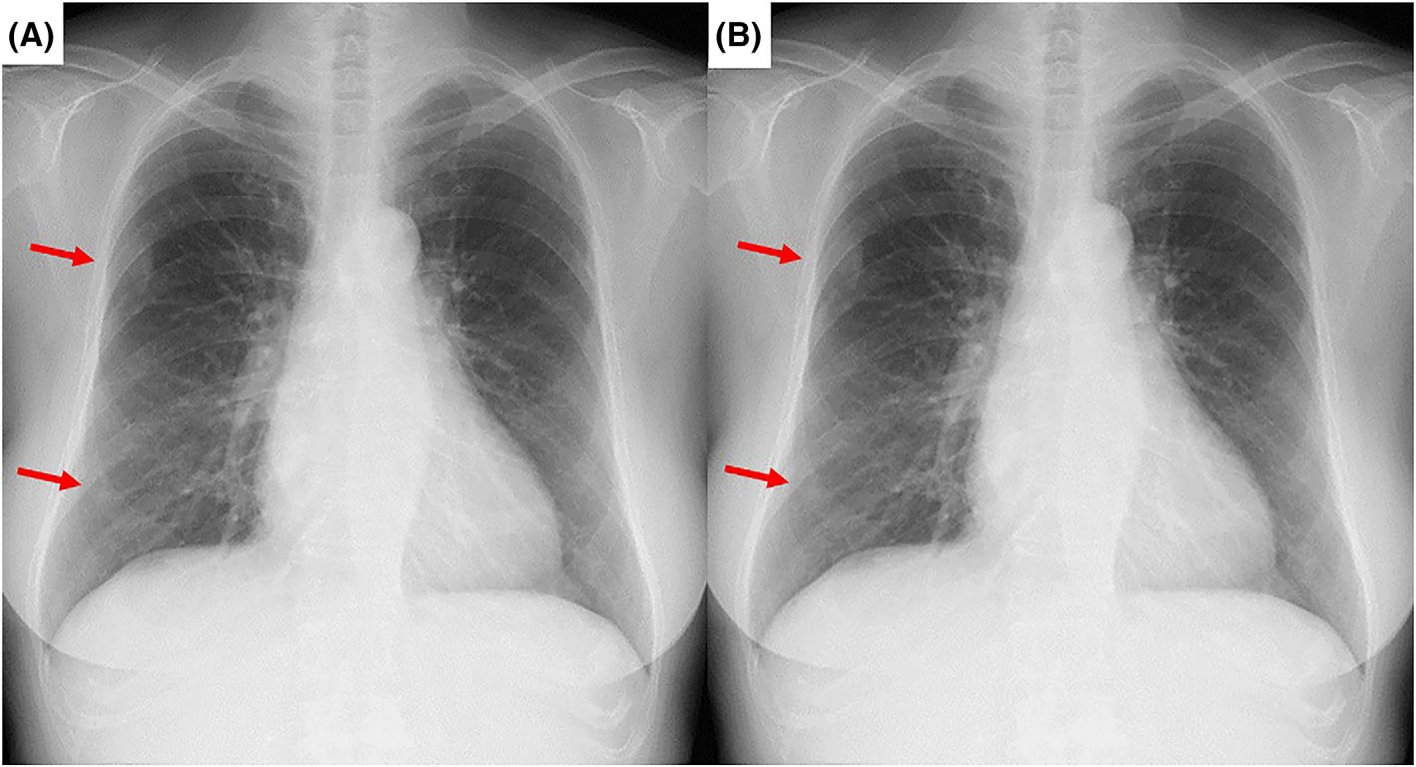
Chest x‐ray images 1 year ago (A) and at present (B) show the nodules in the right lung field. The nodules do not differ significantly during 1 year.

**FIGURE 2:**
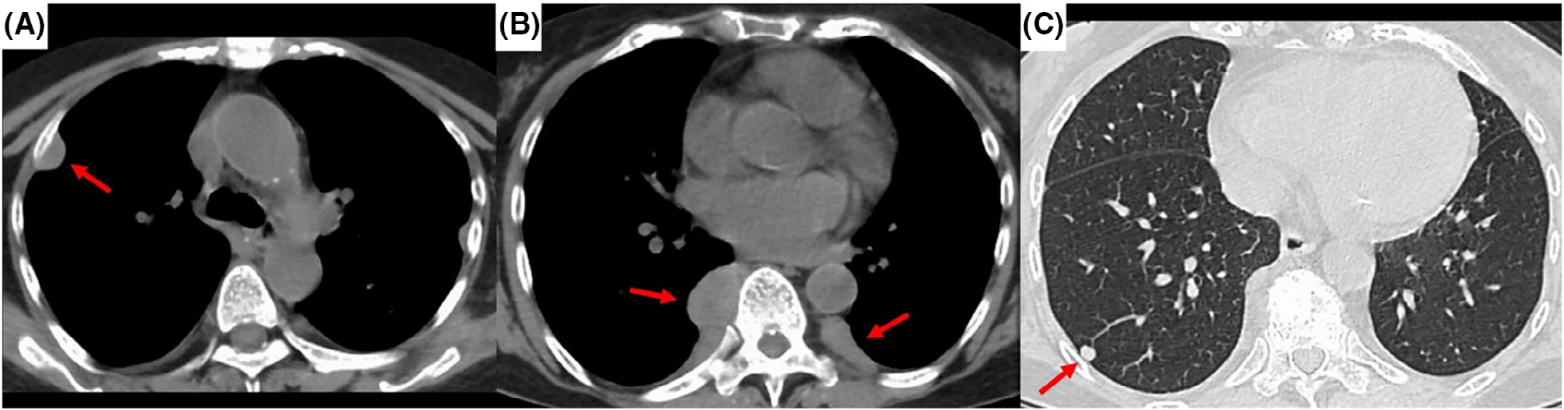
Images of chest computed tomography are shown. In the mediastinal window (A, B), bilateral pleural nodules are revealed. In the pulmonary window (C), a small solid lung nodule is revealed in right lower lobe.

**FIGURE 3:**
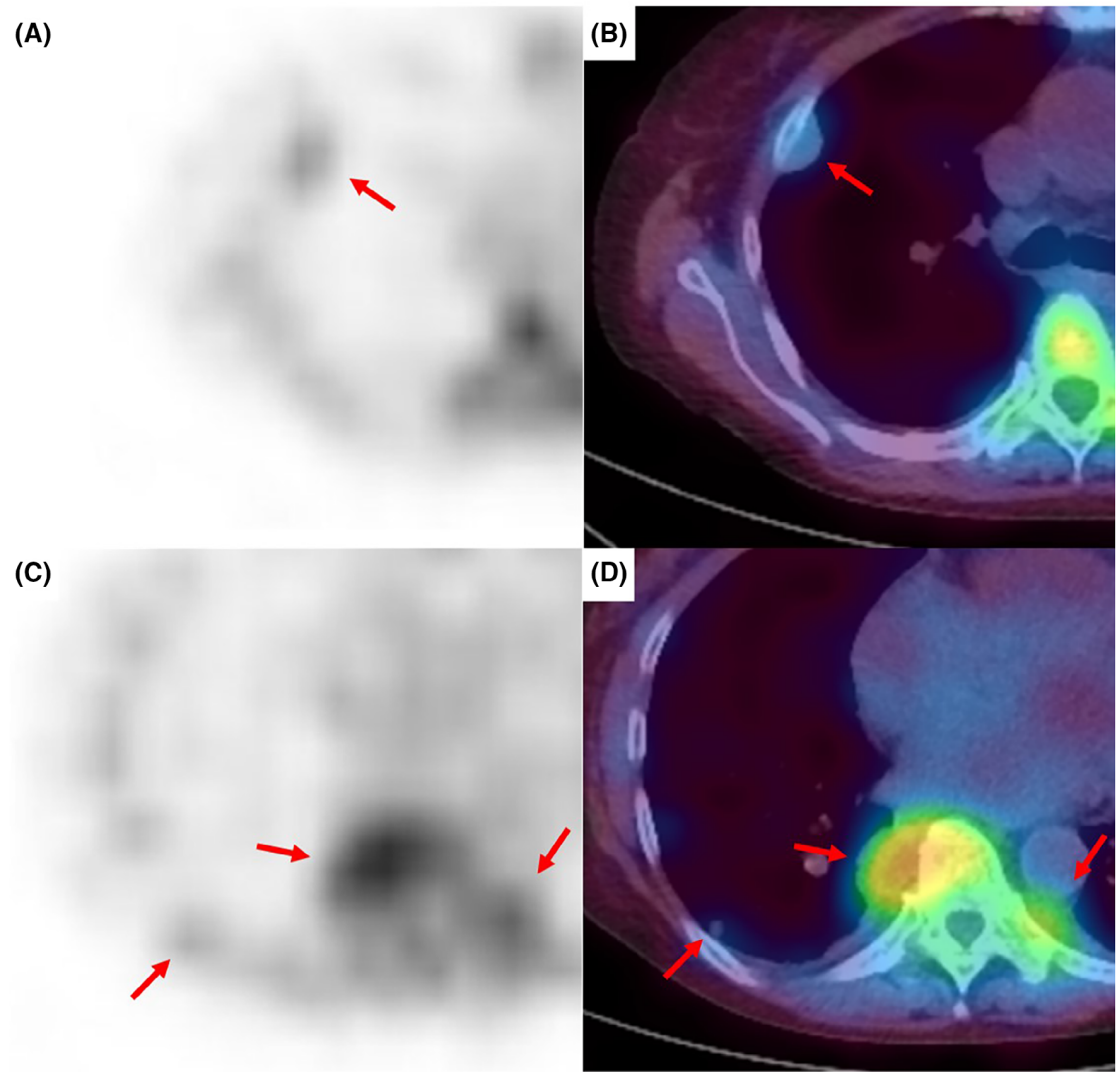
Images of ^111^In‐bone marrow scintigraphy show accumulation of ^111^In in the intrathoracic nodular lesions which locate in the right pleura (A, B), the bilateral paraspinal (C, D), and the right lower lobe (C, D).

A 74‐year‐old female diagnosed with autoimmune hemolytic anaemia was asymptomatic; however, chest x‐ray revealed nodules in the right lung field (Figure 1). Additionally, chest computed tomography (CT) revealed bilateral pleural nodules and a pulmonary nodule in the right lower lobe (Figure 2). ^111^In‐bone marrow scintigraphy was performed, which showed accumulation of ^111^In in the nodular lesions (Figure 3). Based on these findings, the patient was diagnosed with intrathoracic extramedullary haematopoiesis (EMH) without the need for invasive procedures such as CT‐guided needle biopsy and thoracoscopic surgery. We continue to follow the patient, and the lesions have remained unchanged for more than 1 year.

Intrathoracic EMH can present as nodular lesions that need to be differentiated from malignancy. On CT images, pleural lesions of EMH typically form bilateral and multiple masses that have a sharp interface with the lung, especially in the paraspinal location; however, lung lesions of EMH can also present as nonspecific findings.^1^ ^111^In is transported to haematopoietic tissues by transferrin; therefore, extramedullary accumulation of ^111^In can be determined as EMH.^2^ Intrathoracic EMH has a high risk of haemorrhage during biopsy. Therefore, bone marrow scintigraphy should be performed in cases of suspected EMH before needle biopsy or thoracoscopic surgery.

## AUTHOR CONTRIBUTIONS

Kenichiro Takeda collected the clinical data and drafted the original manuscript. Yuki Yato collected the clinical data and supported drafting the original manuscript. Mikihito Saito and Go Saito were in charge of patient care, obtained informed consent from the patient and revised the original manuscript. Akira Nishiyama, Hajime Kasai, and Takuji Suzuki supervised this work and advised on drafting the manuscript. All authors have confirmed the final manuscript and agreed to publication.

## CONFLICT OF INTEREST STATEMENT

None declared.

## ETHICS STATEMENT

The authors declare that appropriate written informed consent was obtained for the publication of this manuscript and accompanying images.

## Data Availability

Data sharing is not applicable to this article as no new data were created or analyzed in this study.
